# Genomic Epidemiology of Hospital‐Associated SARS‐CoV‐2 Clusters in Hong Kong During a Period of Relaxed Visitation (May–August 2022)

**DOI:** 10.1111/irv.70249

**Published:** 2026-03-16

**Authors:** Haogao Gu, Mengting Li, Ruixuan Wang, L. K. Lee, W. Y. Tam, Alex Y. M. Ho, Miranda C. Y. Yau, K. T. Yip, River C. W. Wong, Barry K. C. Wong, Kristine S. Luk, Jimmy Y. W. Lam, T. L. Que, Viola C. Y. Chow, Sandy K. Y. Chau, Sebastian Duchene, Gilman K. H. Siu, Leo L. M. Poon

**Affiliations:** ^1^ School of Public Health, LKS Faculty of Medicine The University of Hong Kong Hong Kong SAR China; ^2^ Department of Health Technology and Informatics The Hong Kong Polytechnic University Hong Kong SAR China; ^3^ Department of Pathology Princess Margaret Hospital Hong Kong SAR China; ^4^ Department of Clinical Pathology Pamela Youde Nethersole Eastern Hospital Hong Kong SAR China; ^5^ Department of Clinical Pathology Tuen Mun Hospital Hong Kong SAR China; ^6^ Department of Microbiology Prince of Wales Hospital Hong Kong SAR China; ^7^ Department of Pathology United Christian Hospital Hong Kong SAR China; ^8^ ED‐ID Unit, Department of Computational Biology Institut Pasteur Paris France; ^9^ Department of Microbiology and Immunology at the Peter Doherty Institute for Infection and Immunity University of Melbourne Melbourne Victoria Australia; ^10^ Centre for Immunology & Infection Hong Kong Science and Technology Park Hong Kong SAR China; ^11^ HKJC Global Health Institute, LKS Faculty of Medicine The University of Hong Kong Hong Kong SAR China; ^12^ HKU‐Pasteur Research Pole, School of Public Health, LKS Faculty of Medicine The University of Hong Kong Hong Kong SAR China

**Keywords:** genomic epidemiology, hospital transmission, nosocomial infections, phylogeography, SARS‐CoV‐2, visitor policy

## Abstract

**Background:**

Between May and August 2022, as Hong Kong shifted from a “zero‐COVID” strategy towards relaxation, including relaxed hospital visitation, understanding SARS‐CoV‐2 nosocomial transmission dynamics was critical.

**Methods:**

We used genomic epidemiology to investigate hospital‐associated infections between May 28 and August 18, 2022. A total of 162 viral genomes from 29 suspected clusters across 17 hospitals were sequenced and analyzed, covering more than half of all officially reported nosocomial infections during the period. Bayesian phylogeographic analysis for the dominant BA.2.2 lineage was used to infer the rate of community‐to‐hospital viral introductions. Generalized additive models (GAMs) then assessed the association between these inferred introduction rates and concurrently measured community and cross‐border mobility indices.

**Results:**

From the initial 29 suspected clusters, 18 nosocomial clusters involving 126 genomes across 13 hospitals were confirmed, representing 51.9% of the total reported nosocomial cases in that period. The dominant lineage was BA.2.2. Most clusters were consistent with single intraward transmission chains, and no evidence of interward spread was found. Bayesian analysis revealed ongoing community‐to‐hospital introductions with a rate that varied considerably over time. However, after accounting for this dominant temporal trend, GAMs found no statistically significant association between the daily rate of these introductions and any of the tested mobility indices.

**Conclusions:**

During periods of sustained community transmission, broad community mobility metrics may not directly predict the risk of SARS‐CoV‐2 introduction into hospitals. Robust hospital‐level infection prevention and control measures, effective visitor screening, and integrated genomic surveillance remain paramount for mitigating nosocomial transmission, irrespective of general population movement.

## 1 Introduction

The COVID‐19 pandemic prompted diverse public health responses worldwide. In Hong Kong SAR (HK), authorities pursued a stringent “zero‐COVID” elimination strategy for nearly 2 years, which effectively suppressed community transmission during HK's first four pandemic waves [1]. Measures included strict border controls, quarantine, and limited social interactions, including near‐complete restrictions on hospital visitation. These nonpharmaceutical interventions (NPIs) were associated with very low rates of nosocomial SARS‐CoV‐2 infection, particularly among healthcare workers, in the early phase [2].

**FIGURE 1:**
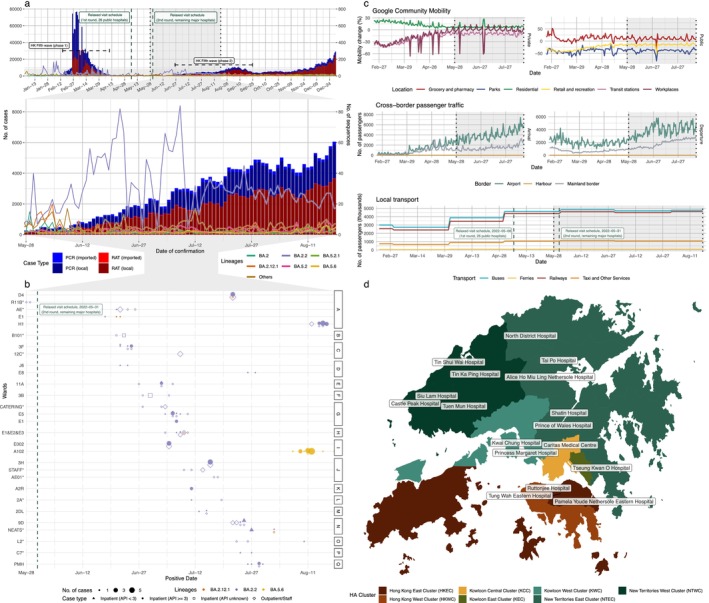
SARS‐CoV‐2 epidemiological landscape, nosocomial clusters, and mobility trends in Hong Kong, May–August 2022. (a) Hong Kong SARS‐CoV‐2 epidemic curve and genomic surveillance. Upper panel: Daily reported COVID‐19 cases (bars, left y‐axis) by detection method (PCR/RAT, local/imported) from January to December 2022. Dashed lines/shaded regions mark hospital visitation relaxation phases (May 6th, May 31st). The study period (May 28th–August 18th) is boxed. Lower panel: Cumulative sequenced genomes and weekly proportions of dominant lineages (colored line, right y‐axis). (b) Characteristics of investigated suspected nosocomial SARS‐CoV‐2 clusters. Each row is a cluster (excluded clusters marked with *. Symbols on x‐axis (Date) show sequenced cases: Size indicates daily case count per cluster, shape denotes case type (Inpatient [API < 3]; Inpatient [API ≥ 3]; Inpatient [API unknown]; Outpatient/Staff), color indicates lineage (blue: BA.2.2; orange: BA.5.6; purple: BA.2.12.1). Right: Hospital abbreviation and administrative HA cluster color (see Panel d). Dashed line: 2nd round visitation relaxation. (c) Population mobility trends, February–August 2022. Google Community Mobility: daily percentage change from prepandemic baseline for various location categories; cross‐border passenger traffic: daily arrivals/departures by border type (airport, harbor, mainland); local transport: daily passenger numbers (thousands) by transport mode. Vertical black dashed lines in all mobility plots indicate the study period. (d) Geographical distribution of hospitals under study. The map shows the locations of hospitals hosting the clusters and samples under study, linked to their respective color‐coded Hospital Authority (HA) administrative cluster (legend provided). Hospital labels shown on the map are anonymized (A–Q) in subsequent analyses and do not identify specific facilities.

This containment period was disrupted by the emergence of highly transmissible Omicron lineages. The fifth wave, peaking in early 2022, overwhelmed previous defenses, leading to an estimated infection of over half of HK's population within approximately 3 months [3, 4], altering the epidemiological landscape and population immunity profile. In May 2022, against a backdrop of declining community prevalence (Figure 1a) and rising population mobility (Figure 1c), the government initiated a phased relaxation of NPIs, including the controlled resumption of hospital visitation—initially in nonacute wards, later extended to acute and specialist hospitals with vaccination and testing requirements for visitors (Table S1).

How ward‐level transmission patterns evolved after visitation resumed remained largely uncharacterized. Prior to May 2022, Hong Kong experienced overall success in preventing hospital‐acquired infection punctuated by isolated outbreaks [5, 6, 7], but the relative contributions of intraward spread, potential interward transmission, and introductions from the community during the postrelaxation period were unclear. Addressing these questions requires high‐resolution genomic data integrated with epidemiological information.

Here, we applied genomic epidemiology to investigate SARS‐CoV‐2 transmission dynamics within Hong Kong public hospitals during May–August 2022. By sequencing viruses from individuals linked to suspected nosocomial clusters and integrating admission timing and ward metadata, we (i) delineate ward‐level transmission clusters, (ii) assess evidence for intraward versus interward spread, and (iii) estimate day‐to‐day community‐to‐hospital introduction pressure. Our observational design evaluates hospital transmission within the context of sustained community circulation under a controlled‐visitation policy.

## 2 Methods

### 2.1 Study Design and Data Collection

We investigated SARS‐CoV‐2 transmission within Hong Kong public hospitals during May 28–August 18, 2022, a period that coincided with the phased relaxation of hospital visitation policies during the fifth epidemic wave (Figure 1a and Table S1). We assembled a genomic‐epidemiologic dataset comprising 162 high‐quality SARS‐CoV‐2 genomes generated from individuals linked to 29 suspected ward‐level clusters in 17 public hospitals. For each sequenced individual, we collated sample collection date, hospital and ward identifiers, and role (inpatient, staff, or outpatient); for inpatients, we additionally obtained admission dates to derive an admission‐to‐positive interval (API).

### 2.2 Case Definitions and Cluster Confirmation

We defined nosocomial infection as hospital‐acquired SARS‐CoV‐2 infection, plausibly transmitted from in‐hospital sources (e.g., an inpatient index) and/or external sources with access to wards (e.g., visitors and healthcare staff with community exposure). During the study period, all inpatients underwent mandatory admission screening for SARS‐CoV‐2 [8], enabling API calculation. We also defined (i) likely nosocomial inpatient: API > 3 days or negative admission screen followed by a positive result with ward exposure, together with genomic linkage to contemporaneous ward cases; and (ii) confirmed nosocomial cluster: ≥ 2 phylogenetically linked cases, including ≥ 1 likely nosocomial inpatient, occurring within the same ward. To determine genomic linkage, we combined pairwise genetic proximity with an ancestral proximity check formulated to distinguish intraward transmission from closely related community introductions circulating at the same time. Full criteria and sensitivity analyses are provided in Supporting Information S1: Methods 5.3.

### 2.3 Sample Collection, Sequencing, and Bioinformatic Processing

Respiratory specimens (combined nasal swabs or deep‐throat saliva) from SARS‐CoV‐2‐positive individuals were processed using the NucliSENS easyMAG platform and screened with the LightMix Sarbeco E‐gene RT‐PCR assay. Whole‐genome sequencing was employed using Oxford Nanopore (MinION/GridION) with the SARS‐CoV‐2 Midnight‐1200 Amplicon Panel and the rapid barcoding protocol (SQK‐RBK110.96). Reads were aligned to the Wuhan‐Hu‐1 reference genome (GenBank MN908947.3) with minimap2, and variants were called with Medaka and Clair to generate consensus sequences. Multiple‐sequence alignment was used with Clustal Omega. Detailed wet‐lab and bioinformatic steps are provided in the Supporting Information S1: Methods.

### 2.4 Epidemiological and Mobility Data Retrieval

To contextualize the genomic data, we summarized officially reported hospital‐related infections (HRI) from the historical news database of the Hospital Authority (HA). We compiled contemporaneous community diagnostic indicators and mobility metrics (Google Community Mobility Reports and governmental reports on cross‐border/local movement) covering the same calendar window. Data curation, preprocessing, and harmonization are described in the Supporting Information.

### 2.5 Phylogenetic and Phylogeographic Analysis

The 162 hospital‐associated sequences were analyzed alongside 2022 Hong Kong public sequences from GISAID (EPI_SET_250322yt). Because Omicron BA.2.2 predominated, we constructed a representative BA.2.2 dataset (*n* = 2092). Time‐scaled Bayesian phylogenies were inferred with BEAST v1.10.5, using a strict molecular clock and Skygrid coalescent model [9]. We implemented discrete trait analysis (DTA) to reconstruct ancestral locations (“community” vs. “hospital”) and estimate transition events between these states. BSSVS identified supported pathways, and Markov jump counting quantified transitions through time [10]. We derived daily community‐to‐hospital introduction rates from the marked phylogenies. Detailed dataset assembly, priors, chain lengths, and diagnostics are provided in Supporting Information S1: Methods 5.1–5.2.

### 2.6 Statistical Analysis of Community‐To‐Hospital Introductions

We assessed correlates of the inferred daily community‐to‐hospital introduction rate using generalized additive models (GAMs) with a negative binomial response. The model included a penalized smooth of time to absorb nonlinear temporal structure; mobility indices were entered as linear terms after variance‐inflation screening (VIF < 5) to mitigate collinearity. We present estimated degrees of freedom (edf), *p*‐values for the smooth, and *p*‐values and incidence rate ratios (IRRs) for mobility predictors, with diagnostics detailed in Supporting Information S1: Methods 6 and Table S4.

### 2.7 Site Labeling and Anonymization

The HA organizes public hospitals into administrative clusters (e.g., Hong Kong East [HKEC], Hong Kong West [HKWC], Kowloon East [KEC], Kowloon Central [KCC], Kowloon West [KWC], New Territories East [NTEC], and New Territories West [NTWC]). For analysis and visualization, individual hospitals were relabeled with neutral codes (A–Q). No patient identifiers (names, personal IDs, and addresses) were collected or retained. Cluster abbreviations are used solely as geographic context. During the study period, nosocomial activity was already reported publicly at the hospital level by the HA; our cluster‐level labels therefore do not newly disclose site identities beyond what was public.

## 3 Results and Discussion

### 3.1 Composition of the Sequenced Cohort and Added Value of the Sequencing Analyses

Across 29 suspected ward‐level clusters spanning 17 public hospitals within multiple HA clusters (Figure 1d), we generated 162 complete genomes from 98 inpatients, 60 staff, and 4 outpatients (Figure 1b). All inpatients underwent admission testing [8], enabling API estimation. The median API was 12.5 days, and 82.2% of inpatients had API ≥ 3 days, consistent with in‐hospital acquisition rather than infection predating admission. A subset of inpatients exhibited very long APIs (300–8591 days), reflecting long‐stay residents in psychiatric facilities who, by design, remain institutionalized for extended periods (Figure S1).

Applying our consistent classification (epidemiology + genomic linkage; Methods; Supporting Information S1: Methods), we identified 18 confirmed nosocomial clusters encompassing 126 genomes across 13 hospitals (Figure 1b and Table S2). These confirmed clusters accounted for ~51.9% of officially reported nosocomial infections during the period (Table S3), underscoring the yield of integrated genomic surveillance for resolving ward transmission. Eleven suspected clusters (36 genomes) were excluded from downstream transmission analyses because phylogenies indicated no direct inpatient linkage and/or clusters comprised staff‐only chains without subsequent inpatient involvement (asterisked in Figure 1b and Table S2).

Whole‐genome sequencing and phylogenetic analysis provided three concrete benefits in this setting. They are (1) discrimination of single‐chain versus multi‐introduction scenarios within wards, even amid the low diversity typical of Omicron, by understanding ancestral proximity and context sequences; (2) exclusion of interward transmission where simultaneous ward activity overlapped in time, thereby supporting the effectiveness of IPC/cohorting measures that limited spread beyond index wards; and (3) quantification of community → hospital introduction pressure by embedding ward sequences within the city‐wide phylogeny and counting Markov jumps, enabling day‐by‐day assessment of importation risk.

### 3.2 Lineage Composition and Within‐Ward/Within‐Hospital Transmission Patterns

During the study window, lineage composition mirrored concurrent community surveillance (Figure 1a): Omicron BA.2.2 dominated, comprising 135/162 (83.4%) genomes and 24/29 initially investigated clusters (Figure 1b). The remainder included 22 BA.5.6 (13.6%) and 5 BA.2.12.1 (3.1%) sequences. The hospital‐focused clades from the full BA.2.2, BA.5.6, and BA.2.12.1 maximum likelihood phylogenetic trees, reconstructed with contemporaneous community public sequences, are shown in Figures S2–S18.

Non‐BA.2.2 phylogenies showed distinct patterns. All BA.5.6 sequences originated from a single ward (cluster A102, Hospital I) and formed a well‐supported monophyletic group alongside public HK BA.5.6 contemporaries, strongly consistent with a single localized chain (Figure S10). BA.2.12.1 was mostly sporadic and polyphyletic (Figures S9 and S15), except for two cases in Hospital A (Figure S2). Given predominance and complexity, subsequent analyses focus on BA.2.2.

Among BA.2.2, 16 of the 18 confirmed clusters showed clear intraward transmission across 13 hospitals (Figures S4–S12, S14–S15, and S18). All (16/16) were compatible with a single inpatient‐involved transmission chain within the ward. To test for intrahospital (interward) spread, we compared phylogenetic distances for temporal overlaps ≤ 10 days among clusters in the same hospital. Despite several overlaps (e.g., E5 and E1 in Hospital G; Figures 1b and S8), we found no phylogenetic support for direct interward transmission linking confirmed clusters. These findings suggest that, within our surveillance coverage, transmission was largely contained within wards, or potential interward connections were too sparse or indirect to resolve given the available data.

### 3.3 Potential Sources of Ward Introductions

Visitor screening and IPC protocols were active throughout the period (Table S1). However, staff with routine community exposure could plausibly seed community‐to‐ward introductions, and our dataset lacked duty rosters/interaction logs and staff longitudinal testing, limiting source attribution. Among the 18 confirmed clusters, we identified two wards (“E1&E2&E3” and “9D”) where ≥ 1 phylogenetically linked staff tested positive before the first likely nosocomial inpatient (Figures 1b, S9, and S15). The remaining 16 confirmed clusters were consistent with a likely inpatient index (API < 3 days) or unknown source (e.g., visitors) initiating the chain.

### 3.4 Community–Hospital Interface for BA.2.2

**FIGURE 2:**
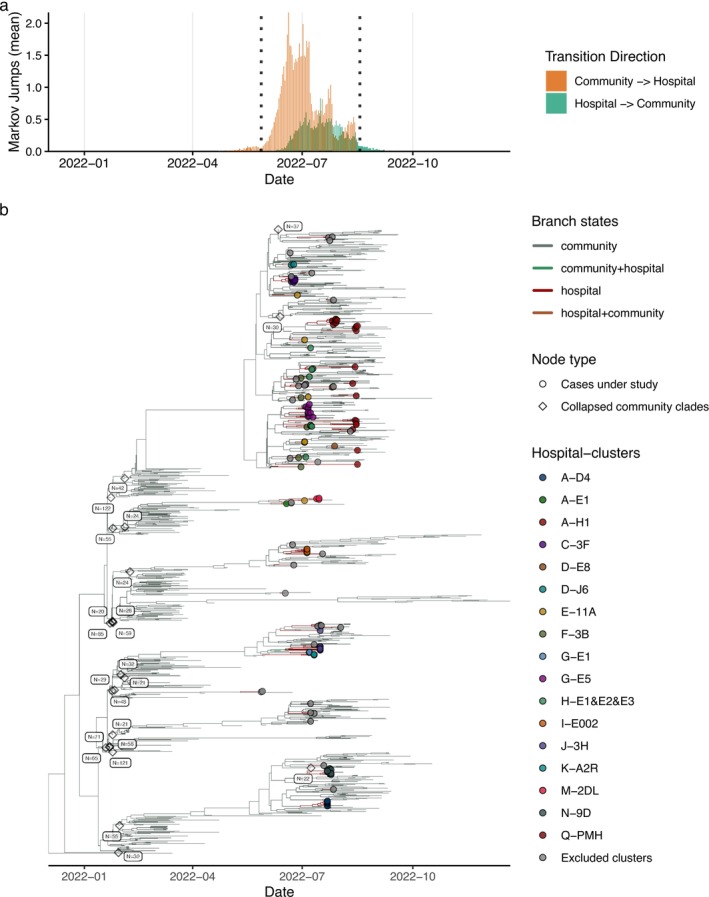
Phylogeographic inference of SARS‐CoV‐2 BA.2.2 transmission dynamics between community and hospital settings in Hong Kong. (a) Daily mean number of inferred Markov jumps between community and hospital states. The y‐axis shows the mean number of jumps across the posterior tree distribution. Orange areas represent transitions from community to hospital, and teal areas represent transitions from hospital to community. Data are plotted from January to October 2022, with the primary peak of transitions occurring during the study period (May–August 2022). Vertical black dashed lines indicate the study period. (b) Time‐scaled Bayesian phylogeny of Hong Kong BA.2.2 sequences, annotated with ancestral location states and hospital cluster membership. The x‐axis represents time (January to October 2022). Branches are colored according to their inferred ancestral state: grey (community), red (hospital), green (community + hospital, indicating transitions on short branches or uncertainty), brown (hospital + community). Tip nodes representing sequences from the 16 confirmed hospital clusters included in this study are shown as solid colored circles, with colors corresponding to individual hospital clusters as defined in the “Hospital‐clusters” legend (e.g., A‐D4). Tip nodes representing sequences from excluded clusters are shown as smaller, dark grey circles. Uncolored open diamonds represent collapsed community clades (with the number of descendant tips [e.g., *N* = 122]) not directly part of the investigated hospital clusters or individual community sequences.

Using DTA with Markov jump counting, we inferred directional transitions between community and hospital states on time‐scaled phylogenies (Methods). Over May–August 2022, the analysis indicated more community → hospital than hospital → community transitions (Figure 2), consistent with ongoing importation pressure into wards during community Omicron activity. As with any DTA, inference is sensitive to sampling (particularly underrepresentation of community sequences not associated with hospital investigations) and to the accuracy of state annotations for older nodes [11]. These limitations motivate cautious interpretation of absolute counts while supporting relative patterns through time.

### 3.5 Mobility Correlates of Inferred Introductions

We modeled the daily rate of inferred community → hospital introductions against concurrent mobility indices using GAMs (negative binomial), including a time smooth to account for nonlinear temporal structure (Methods; Supporting Information S1: Methods 6). After VIF screening (VIF < 5), eight predictors were retained (Table S4).

The model yielded a significant temporal smooth (edf = 2.83, *p* = 0.027), indicating that introduction risk varied systematically over time, likely tracking community prevalence and other unmeasured temporal factors. None of the eight mobility predictors showed a statistically significant independent association with introduction rate after controlling for time (all *p* > 0.5); 95% CIs for all predictor IRRs spanned 1.0 (Table S4). The model's Adjusted *R* ^2^ = 0.798 and Deviance Explained = 42.7% (Figure S19) indicate that the time component captured the major share of explainable variation, whereas daily fluctuations in the specific mobility metrics contributed little additional signal.

We did not detect associations between common mobility indicators and inferred introductions during this window, despite resumed visitation and broader population movement. Several factors are plausible: (i) Community transmission intensity (absorbed by the time smooth) likely dominated daily importation risk; (ii) the granularity of aggregate mobility indices may miss ward‐relevant exposures (e.g., visitor throughput to specific wards and healthcare‐worker community contacts); and (iii) mitigation measures linked to visitation (screening) plus routine hospital IPC may have tempered any marginal effect of general mobility on hospital importations.

### 3.6 Broader Context and Limitations

First, our multihospital genomic surveillance during this transition period helps fill a previous gap: Before May 2022, Hong Kong lacked large‐scale, systematic WGS focused on nosocomial transmission across multiple hospitals, limiting baseline comparisons with the pre‐relaxation period. Although WGS had been used to track community transmission and discrete outbreaks [5, 7], the hospital‐focused network structure during the “zero‐COVID” era was not comprehensively characterized, making it difficult to attribute any change solely to visitation policy versus high community Omicron prevalence and evolving sublineages.

Second, our cluster confirmation rests on integrated epidemiologic–genomic criteria that have inherent limitations under short timeframes and low viral diversity. That said, we achieved high nosocomial sampling intensity, which should reduce sampling bias and provide a more representative view of ward transmission than studies with sparse coverage. DTA‐based Markov jumps are informative but sampling‐sensitive; we therefore emphasize relative temporal patterns rather than absolute counts alone [11].

Third, source apportionment remains uncertain: We lacked duty rosters, interaction logs, and staff longitudinal testing, so we cannot definitively partition introductions among visitors, staff, and other contacts. Given routine community interaction by healthcare workers during high incidence, staff‐mediated seeding is plausible. This argues for sustained staff IPC and surveillance alongside visitor screening.

Finally, we acknowledge the well‐documented benefits of visitation for patient well‐being and clinical outcomes [12]. Policy must balance these benefits against infection risk. Our results suggest that targeted IPC and ongoing genomic surveillance can help minimize ward spread without banning all visitation.

## 4 Conclusion

In this multihospital study spanning May–August 2022, we integrated genomics and epidemiology to characterize 18 confirmed nosocomial clusters (126 genomes) drawn from 162 sequences linked to 29 suspected clusters in 17 public hospitals. Intraward transmission predominated, with no phylogenetic evidence for interward spread among contemporaneous clusters within the same hospital. DTA/Markov jumps indicated sustained community → hospital introductions, but GAMs found no independent association with concurrent mobility indices after accounting for temporal trends, implying that background community prevalence was the primary driver of introduction risk during this period. Together, these findings support robust IPC, visitor screening, and continuous, integrated genomic surveillance as practical levers to manage nosocomial threats while preserving the benefits of visitation.

## Author Contributions

**Haogao Gu:** methodology, data curation, formal analysis, visualization, writing – original draft, and writing – review and editing. **Mengting Li:** data curation and writing – review and editing. **Ruixuan Wang:** methodology, formal analysis, and writing – review and editing. **L. K. Lee:** investigation, data curation, and writing – review and editing. **W. Y. Tam:** investigation, data curation, and writing – review and editing. **Alex Y. M. Ho:** resources and writing – review and editing. **Miranda C. Y. Yau:** resources and writing – review and editing. **K. T. Yip:** resources and writing – review and editing. **River C. W. Wong:** resources and writing – review and editing. **Barry K. C. Wong:** resources and writing – review and editing. **Kristine S. Luk:** resources and writing – review and editing. **Jimmy Y. W. Lam:** resources and writing – review and editing. **T. L. Que:** resources and writing – review and editing. **Viola C. Y. Chow:** resources and writing – review and editing. **Sandy K. Y. Chau:** resources and writing – review and editing. **Sebastian Duchene:** methodology and writing – review and editing. **Gilman K. H. Siu:** conceptualization, resources, funding acquisition, supervision, and writing – review and editing. **Leo L. M. Poon:** conceptualization, resources, funding acquisition, supervision, and writing – review and editing.

## Funding

This work was supported by the Health and Medical Research Fund (COVID190204), Research Grants Council of HK theme‐based research schemes (T11‐705/21‐N), InnoHK C2i, and Hong Kong Jockey Club Charities Trust (HKJCGHI).

## Conflicts of Interest

The authors declare no conflicts of interest.

## Supporting information


**Table S1:** Relaxation of hospital visitation policies in Hong Kong (Post‐May 2022).
**Table S2:** Details of hospital SARS‐CoV‐2 clusters sampled for investigation, including epidemiological data and classification rationale (May 28–August 18, 2022).
**Table S3:** Summary of nosocomial SARS‐CoV‐2 infections officially reported by the Hong Kong Hospital Authority (May 28–August 18, 2022) (no mapping is provided between named HA press items and the anonymized A–Q codes).
**Table S4:** Generalized additive models (GAM) model results summary.
**Figure S1:** The distribution of admission‐to‐positive interval (API) for inpatient cases in this study. The inner plot is a zoom view of the case distribution between API of 0–80.
**Figure S2:** Extracted clade from the full reconstructed BA.2.2 and BA.2.12.1 maximum likelihood phylogenetic trees, focusing on sequences from Hospital A. Focused tips are labeled; Branch supports for ancestral nodes of focused sequences are labeled in blue.
**Figure S3:** Extracted clade from the full reconstructed BA.2.2 maximum likelihood phylogenetic tree, focusing on sequences from Hospital B. Focused tips are labeled; Branch supports for ancestral nodes of focused sequences are labeled in blue.
**Figure S4:** Extracted clade from the full reconstructed BA.2.2 maximum likelihood phylogenetic tree, focusing on sequences from Hospital C. Focused tips are labeled; Branch supports for ancestral nodes of focused sequences are labeled in blue.
**Figure S5:** Extracted clade from the full reconstructed BA.2.2 maximum likelihood phylogenetic tree, focusing on sequences from Hospital D. Focused tips are labeled; Branch supports for ancestral nodes of focused sequences are labeled in blue.
**Figure S6:** Extracted clade from the full reconstructed BA.2.2 maximum likelihood phylogenetic tree, focusing on sequences from Hospital E. Focused tips are labeled; Branch supports for ancestral nodes of focused sequences are labeled in blue.
**Figure S7:** Extracted clade from the full reconstructed BA.2.2 maximum likelihood phylogenetic tree, focusing on sequences from Hospital F. Focused tips are labeled; Branch supports for ancestral nodes of focused sequences are labeled in blue.
**Figure S8:** Extracted clade from the full reconstructed BA.2.2 maximum likelihood phylogenetic tree, focusing on sequences from Hospital G. Focused tips are labeled; Branch supports for ancestral nodes of focused sequences are labeled in blue.
**Figure S9:** Extracted clade from the full reconstructed BA.2.2 and BA.2.12.1 maximum likelihood phylogenetic trees, focusing on sequences from Hospital H. Focused tips are labeled; Branch supports for ancestral nodes of focused sequences are labeled in blue.
**Figure S10:** Extracted clade from the full reconstructed BA.2.2 and BA.5.6 maximum likelihood phylogenetic trees, focusing on sequences from Hospital I. Focused tips are labeled; Branch supports for ancestral nodes of focused sequences are labeled in blue.
**Figure S11:** Extracted clade from the full reconstructed BA.2.2 maximum likelihood phylogenetic tree, focusing on sequences from Hospital J. Focused tips are labeled; Branch supports for ancestral nodes of focused sequences are labeled in blue.
**Figure S12:** Extracted clade from the full reconstructed BA.2.2 maximum likelihood phylogenetic tree, focusing on sequences from Hospital K. Focused tips are labeled; Branch supports for ancestral nodes of focused sequences are labeled in blue.
**Figure S13:** Extracted clade from the full reconstructed BA.2.2 maximum likelihood phylogenetic tree, focusing on sequences from Hospital L. Focused tips are labeled; Branch supports for ancestral nodes of focused sequences are labeled in blue.
**Figure S14:** Extracted clade from the full reconstructed BA.2.2 maximum likelihood phylogenetic tree, focusing on sequences from Hospital M. Focused tips are labeled; Branch supports for ancestral nodes of focused sequences are labeled in blue.
**Figure S15:** Extracted clade from the full reconstructed BA.2.2 and BA.2.12.1 maximum likelihood phylogenetic trees, focusing on sequences from Hospital N. Focused tips are labeled; Branch supports for ancestral nodes of focused sequences are labeled in blue.
**Figure S16:** Extracted clade from the full reconstructed BA.2.2 maximum likelihood phylogenetic tree, focusing on sequences from Hospital O. Focused tips are labeled; Branch supports for ancestral nodes of focused sequences are labeled in blue.
**Figure S17:** Extracted clade from the full reconstructed BA.2.2 maximum likelihood phylogenetic tree, focusing on sequences from Hospital P. Focused tips are labeled; Branch supports for ancestral nodes of focused sequences are labeled in blue.
**Figure S18:** Extracted clade from the full reconstructed BA.2.2 maximum likelihood phylogenetic tree, focusing on sequences from Hospital Q. Focused tips are labeled; Branch supports for ancestral nodes of focused sequences are labeled in blue.
**Figure S19:** Estimated smooth function of the GAM model with the fitted data.

## Data Availability

The data that support the findings of this study are openly available on Github at https://github.com/Leo‐Poon‐Lab/GenEpi‐HRI‐HK‐2022.
