# Supplementary material for: Genomic Epidemiology of Hospital‐Associated SARS‐CoV‐2 Clusters in Hong Kong During a Period of Relaxed Visitation (May–August 2022)
Source: Influenza Other Respir Viruses. 2026 Mar 16;20(3):e70249. doi: 10.1111/irv.70249 (PMC13097321; doi:10.1111/irv.70249)
Supplement: Supplementary file 1 — Table S1: Relaxation of hospital visitation policies in Hong Kong (Post‐May 2022). Table S2: Details of hospital SARS‐CoV‐2 clusters sampled for investigation, including epidemiological data and classification rationale (May 28–August 18, 2022). Table S3: Summary of nosocomial SARS‐CoV‐2 infections officially reported by the Hong Kong Hospital Authority (May 28–August 18, 2022) (no mapping is provided between named HA press items and the anonymized A–Q codes). Table S4: Generalized additive models (GAM) model results summary. Figure S1: The distribution of admission‐to‐positive interval (API) for inpatient cases in this study. The inner plot is a zoom view of the case distribution between API of 0–80. Figure S2: Extracted clade from the full reconstructed BA.2.2 and BA.2.12.1 maximum likelihood phylogenetic trees, focusing on sequences from Hospital A. Focused tips are labeled; Branch supports for ancestral nodes of focused sequences are labeled in blue. Figure S3: Extracted clade from the full reconstructed BA.2.2 maximum likelihood phylogenetic tree, focusing on sequences from Hospital B. Focused tips are labeled; Branch supports for ancestral nodes of focused sequences are labeled in blue. Figure S4: Extracted clade from the full reconstructed BA.2.2 maximum likelihood phylogenetic tree, focusing on sequences from Hospital C. Focused tips are labeled; Branch supports for ancestral nodes of focused sequences are labeled in blue. Figure S5: Extracted clade from the full reconstructed BA.2.2 maximum likelihood phylogenetic tree, focusing on sequences from Hospital D. Focused tips are labeled; Branch supports for ancestral nodes of focused sequences are labeled in blue. Figure S6: Extracted clade from the full reconstructed BA.2.2 maximum likelihood phylogenetic tree, focusing on sequences from Hospital E. Focused tips are labeled; Branch supports for ancestral nodes of focused sequences are labeled in blue. Figure S7: Extracted clade from the full reconstructed BA.2 [file IRV-20-e70249-s001.docx]

Supplementary Methods

1. **Diagnostic surveillance data**

To understand the broader epidemiological context and official reporting landscape during the study period (May 28th – August 18th, 2022), we collected community-level and hospital-specific surveillance data from two primary public sources.

Firstly, aggregated daily statistics for Hong Kong, including confirmed COVID-19 case classifications (e.g., differentiating local vs. imported cases) and the reported number of SARS-CoV-2 samples sequenced, were retrieved from the comprehensive COVID-19 dashboard maintained by the University of Hong Kong's School of Public Health (Source: https://covid19.sph.hku.hk/). Data corresponding to the 2022 study period were programmatically curated from the website using custom in-house scripts for subsequent analysis and visualization. The code used for data curation and plotting is available at <https://github.com/Leo-Poon-Lab/GenEpi-HRI-HK-2022> . This provided context on overall community transmission intensity and the scale of concurrent genomic surveillance efforts.

Secondly, specific information regarding officially reported hospital-related COVID-19 infections was systematically gathered from the Hong Kong Hospital Authority (HA) website. We performed searches within the HA's archived press release database (Source: https://www.ha.org.hk/visitor/ha_visitor_index.asp?Content_ID=643&Lang=ENG&Dimension=100&Parent_ID=10000) to identify all relevant daily announcements published during the study timeframe. These press releases, typically titled along the lines of "Hospital Authority announces positive patient cases detected via admission screening or testing and clusters of nosocomial COVID-19 infections," contain the official public reporting of cases identified within HA facilities. We manually curated data from these releases, specifically extracting the counts of patients identified as positive via admission screening (AS) and those classified as nosocomial infection (NI) cases, along with any reported details on nosocomial infection clusters. This dataset provided the official public health reporting context for hospital-associated infections during the period investigated.

1. **Mobility data**

To evaluate potential associations between community movement patterns and hospital infection dynamics during the study period (May 28th – August 18th, 2022), population mobility data were collated from three distinct, publicly available sources.

1. Google Community Mobility Reports (GMR): Anonymized, aggregated data were obtained from Google LLC (Source: https://www.google.com/covid19/mobility/index.html?hl=en). These reports quantify daily movement trends as percentage changes relative to a pre-pandemic baseline period (median value, for the corresponding day of the week, during Jan 3 – Feb 6, 2020). Data are categorized by location type, including retail and recreation, grocery and pharmacy, parks, transit stations, workplaces, and residential areas, providing insights into overall community activity levels and adherence to potential restrictions.
2. Governmental Local Mobility Data: Statistics regarding intra-city transport usage within Hong Kong were sourced from the Hong Kong Transport Department's "Monthly Traffic and Transport Digest" publications corresponding to the study months in 2022 (Example structure/access point: https://www.td.gov.hk/en/transport_in_hong_kong/transport_figures/monthly_traffic_and_transport_digest/). Data extracted typically include figures such as daily average patronage across major public transport operators (e.g., MTR railways, franchised buses) and daily average vehicular traffic through major cross-harbour tunnels, offering quantitative measures of internal movement volume.
3. Governmental Cross-Border Mobility Data: Daily statistics on passenger traffic flow into and out of Hong Kong were retrieved from the Hong Kong Immigration Department, accessed via the government's public sector information portal (Source: https://data.gov.hk/en-data/dataset/hk-immd-set5-statistics-daily-passenger-traffic). This dataset details the number of arrivals and departures through various immigration control points (e.g., Airport, Lo Wu, Lok Ma Chau Spur Line, Hong Kong-Zhuhai-Macao Bridge), often stratified by resident status, providing a measure of external population movement.

Key metrics derived for analysis included total daily local public transport passengers, daily arrivals/departures via Airport and Mainland border control points, and Google mobility indices for parks, grocery/pharmacy, and workplaces.

1. **Sample collection, laboratory diagnosis and whole-genome sequencing**

Respiratory specimens (nasal swabs or deep throat saliva) were collected from patients. Total nucleic acid was extracted from these samples using the NucliSENS® easyMAG® (bioMérieux, The Netherlands) following the standard protocol from the manufacturers. Extracted RNA was tested for SARS-CoV-2 using the TIB-Molbiol LightMix® SarbecoV E-gene assay (TIB-MolBiol, Berlin, Germany) as previously described (1). For whole-genome sequencing (WGS), RNA elutes were first treated with the Turbo DNA-free Kit (ThermoFisher Scientific, Waltham, MA, USA) to remove host DNA. Subsequently, cDNA was synthesized using the LunaScript RT SuperMix Kit (New England BioLabs Inc. (NEB), Hitchin, UK). The cDNA was then amplified via multiplex PCR employing Q5 Hot Start High-Fidelity 2X Master Mix (NEB) using the SARS-CoV-2-Midnight-1200 Amplicon Panel (Integrated DNA Technologies, Coralville, IA, USA). The amplicons were quantified using the Qubit 2 fluorometer (Thermo Fisher Scientific, US) prior to library preparation. The library preparation was performed following the protocol ‘PCR tiling of SARS-CoV-2 virus - rapid barcoding (SQK-RBK110.96)’ (version: PCTR_9125_v110_revH_24Mar2021-minion). The library was loaded and whole-genome seqeuenced on MinION or GridION (Oxford Nanopore Technologies, UK) with R9.4.1 flow cells.

1. **Bioinformatic processing of sequencing data**

Bioinformatic analysis of the sequencing data was conducted using pipelines adapted from the ARTIC network's nCoV-2019 protocol (https://artic.network/ncov-2019/ncov2019-it-setup.html). Briefly, reads were filtered to retain lengths between 400 and 700 bp. These filtered reads were then mapped to the Wuhan-Hu-1 reference genome (GenBank: MN908947.3) using minimap2 (v2.17; <https://anaconda.org/bioconda/minimap2>). Variant calling to generate consensus sequences was performed using both the Medaka (<https://github.com/nanoporetech/medaka>) and Clair (<https://github.com/HKU-BAL/Clair>) pipelines. Nucleotide substitutions were numbered relative to the Wuhan-Hu-1 reference. Finally, multiple sequence alignment of the consensus genomes was performed using Clustal Omega (v1.2.0; <http://www.clustal.org/omega/>).

1. **Phylogenetic and phylogeographic analysis**

*5.1. Dataset Assembly and Time-Resolved Phylogeny Reconstruction*

All SARS-CoV-2 hospital sequences generated in this study (n=162) were analyzed alongside public sequences from Hong Kong collected in 2022, retrieved from the GISAID database (2) on 25 March 2025 (GISAID Identifier: EPI_SET_250322yt, DOI: 10.55876/gis8.250322yt). For the dominant BA.2.2 lineage, the public dataset was subsampled monthly proportionally to the community epidemiological curve to ensure representativeness while maintaining computational feasibility, resulting in a final BA.2.2 dataset (n=2,089) comprising 135 hospital sequences and 1,954 public sequences. For the minor lineages BA.2.12.1 (n=436 total, 5 hospital) and BA.5.6 (n=386 total, 22 hospital), all available contemporaneous public sequences were included.

Multiple sequence alignments were generated using MAFFT v7.520 (3). Maximum likelihood (ML) phylogenetic trees were constructed using IQ-TREE v2.2.5 (4) under the GTR+G model. Branch support was assessed using 1,000 ultrafast bootstrap replicates (UFBoot (5)) and 1,000 SH-like approximate likelihood ratio test replicates (SH-aLRT (6)). Initial time-scaled phylogenies were inferred from the ML trees using TreeTime v0.11.1 (7), estimating substitution rates via root-to-tip regression and resolving polytomies randomly.

Temporal signal was assessed using TempEst v1.5.3 (8) prior to Bayesian phylogenetic analysis. We set up the phylogenetic model using a pre-release of BEAST v.1.10.5 (Thorney BEAST v0.1.1 (9), https://beast.community/thorney_beast), which utilizes a simplified Poisson likelihood for computational efficiency (10, 11). A strict molecular clock model was applied, with the clock rate prior specified as a log-normal distribution. Population dynamics were modelled using a Skygrid coalescent prior with weekly grid points (12). Input trees were derived from both ML and initial TreeTime inferences. Four independent MCMC chains were run for 5 × 10^8^ steps each, sampling every 1 × 10^5^ steps. Convergence was assessed in Tracer v1.7.2, ensuring effective sample sizes (ESS) > 100 for key parameters after discarding the initial 10% as burn-in. Trees from replicate runs were combined, and a representative subset of 600 posterior trees was retained for subsequent analyses after appropriate thinning.

- 1. *Phylogeographic Discrete Trait Analysis (DTA)*

To reconstruct ancestral locations and estimate viral transitions, a Bayesian discrete phylogeographic analysis was performed using the DTA model implemented in BEAST v1.10.5. Sequences were assigned discrete location states: "community" or "hospital". An asymmetric substitution model was employed, allowing for different forward and backward transition rates between states. Bayesian Stochastic Search Variable Selection (BSSVS) was used to identify statistically supported transition pathways among locations. Markov jump counting was performed across the posterior tree set using the `TreeMarkovJumpHistoryAnalyzer` tool (13) to estimate the number and timing of transitions along phylogenetic branches.

The output from `TreeMarkovJumpHistoryAnalyzer` provides the estimated time (in decimal years) for each inferred Markov jump event on each posterior tree. To obtain daily rates for specific transitions (e.g., community-to-hospital), these jump timings were first converted to calendar dates. For each day within the study period (May 28th to August 18th, 2022), the total number of inferred community-to-hospital jumps occurring on that day was summed across all 501 posterior trees. This sum was then divided by the total number of posterior trees to yield the average estimated daily rate (`MJ_value`) for that specific transition, representing the mean posterior expectation of the jump rate for that day.

- 1. *Methodology for identifying phylogenetically linked ward clusters*

**Objective:** To identify clusters of viral sequences from epidemiologically linked individuals (e.g., patients and staff within a specific hospital ward – "Epi-linked Tips") suggestive of ward-based transmission, by assessing their phylogenetic relationship relative to contemporaneous sequences from the wider community ("Community Tips"). This method specifically aims to avoid clustering epi-linked sequences if a more closely related ancestral community sequence exists, which would suggest independent introductions rather than a single transmission chain. Also note that we excluded clusters without sufficient metadata supporting inpatient involvement. It is because establishing the infection setting (hospital vs. community) for staff-only clusters remains challenging given staff mobility and the typically shared genetic background of circulating viruses during periods of sustained community transmission.

**Definitions:**

- **Epi-linked Tips:** Tips representing sequences from the specific epidemiologically linked group.
- **Community Tips:** Tips representing sequences *not* part of the specific epi-linked group.
- **MRCA:** Most Recent Common Ancestor.
- **Patristic Distance:** Sum of branch lengths connecting two nodes.
- **Initial Core Cluster:** A minimal group composed two or more Epi-linked Tips sharing an immediate common ancestor node. If there is no such group, every individual Epi-linked Tip will become an Initial Core Cluster.
- **Candidate Linked Tip (CLT):** An Epi-linked Tip collected being evaluated for inclusion with an existing cluster based on potential ancestral relationship.

**Procedure:**

1. **Identify Initial Core Clusters:** Define all Initial Core Clusters as described above. These represent the most direct evidence of shared ancestry within the epi-linked group.
2. **Evaluate Potential Ancestral Links:** For each cluster (starting with Initial Core Clusters), identify its MRCA (MRCA_cluster). Consider any single Epi-linked Tip (CLT) that connects to an ancestral node (Ancestral_Node) to MRCA_cluster within the phylogeny being evaluated.
3. **Cluster Expansion Criteria - The Ancestral Proximity Check:** A CLT is merged into the cluster descending from MRCA_cluster *only if* the following condition is met:
   - There is *no* Community Tip (Community_Tip) anywhere in the phylogeny that simultaneously satisfies **both** of the following:
     - **(a) Ancestral Relationship:** The Community_Tip diverges from a node that is descendant to Ancestral_Node, and the node divergence should represent an earlier split relative to the MRCA_cluster divergence. Essentially, the Community_Tip is not a direct descendant *within* the cluster being formed or expanded.
     - **(b) Closer Proximity:** The patristic distance from this Community_Tip to MRCA_cluster is *less* than the patristic distance from the CLT to MRCA_cluster.
   - *Rationale:* This criterion prevents the merging of a CLT with a cluster if there is evidence of a closer phylogenetic link between the cluster and a more ancestral community sequence. Such a finding would suggest the cluster members and the CLT might represent independent introductions from different points within that broader community lineage, rather than direct transmission between the CLT and the cluster members. This approach specifically does *not* preclude merging if *sister* Community Tips (diverging from the same Parent_Node) are equally close, as this scenario is compatible with a single introduction event followed by parallel transmission in both ward and community settings.
4. **Node Support:** Reasonable statistical support (see above) for the Parent_Node and MRCA_cluster involved in the potential linkage are required before merging.
5. **Iterative Application:** If a CLT is merged, the expanded group becomes the new cluster. Repeat steps 2-4, moving progressively up the tree until no further CLTs meet the criteria for merging with existing clusters.
6. **Final Cluster Definition:** The resulting groups are defined as the "Phylogenetically Linked Ward Clusters".
7. **Statistical Analysis of Community Introductions (GAM)**

*6.1. Response and Predictor Variables*

The primary response variable for the statistical modelling was the estimated daily rate of community-to-hospital Markov jumps (`MJ_value`), calculated as described in section 5.2. Potential predictor variables included daily time series data obtained from the sources detailed in Section 2.

*6.2. Collinearity Assessment and Predictor Selection*

Multicollinearity among the potential predictor variables was assessed prior to GAM fitting. Pairwise Pearson correlation coefficients were calculated for all predictors. Variance Inflation Factors (VIFs) were calculated using the `vif` function from the `car` package in R (version 4.2.2), applied to a temporary linear model including all potential predictors. Predictors exhibiting high collinearity (defined as VIF > 5) were considered for removal or aggregation. Based on this assessment (details in R code comments), the final set of predictors included in the GAM analysis comprised: total daily local public transport passengers, daily cross-border arrivals and departures via Airport, daily cross-border arrivals and departures via Mainland border control points, and Google mobility indices for Parks, Grocery & Pharmacy, and Workplaces. All selected predictors had VIF values below 5 in the final model check.

6.3. Generalized Additive Model (GAM) Specification

A Generalized Additive Model (GAM) was fitted to the daily time series data using the `gam` function within the `mgcv` package in R. The model aimed to predict the daily community-to-hospital jump rate (`MJ_value`) based on the selected concurrent predictors while accounting for temporal dependencies. The model formula was specified as:

`MJ_value ~ s(time_index, bs = "cr", k = K) + predictor1 + predictor2 + ...`

where `time_index` represents the sequential day number (1 to 83) within the study period. We used a **cubic regression spline** basis (**bs='cr'**) for the time smooth (k=20). The remaining terms represent the linear effects of the selected, concurrent predictor variables identified in section 6.2.

A Negative Binomial distribution (`family = nb()`) with a log link function was specified to model the response variable, accommodating potential overdispersion commonly found in count-like data. The model was fitted using Restricted Maximum Likelihood (`method = "REML"`).

6.4. Model Checking and Interpretation

Model adequacy was assessed using standard diagnostic tools provided by the `mgcv` package. The `summary(gam_model)` output provided estimates, standard errors, z-values, and p-values for parametric coefficients (predictors) and information on the estimated degrees of freedom (edf) and significance of the smooth term. The `gam.check()` function was used to evaluate residual patterns, check the adequacy of the basis dimension `k` for the smooth term, and assess the appropriateness of the Negative Binomial distribution (e.g., via QQ-plots). Autocorrelation functions (ACF) and partial autocorrelation functions (PACF) of the model residuals were examined using the `forecast` package to check for remaining temporal dependence. Parametric coefficients were interpreted as log-incidence rate ratios, and exponentiated coefficients (Incidence Rate Ratios - IRRs) along with their 95% confidence intervals were calculated for ease of interpretation.

Supplementary Tables and Figures

**Table S1.** Relaxation of Hospital Visitation Policies in Hong Kong (Post-May 2022)

| **Phase** | **Commencement Date** | **Scope (Hospital Types/Wards)** | **Visitor Frequency/Duration** | **Vaccination Requirements** | **Testing Requirements** | **Other Key Conditions** |
| --- | --- | --- | --- | --- | --- | --- |
| 1 | May 6th, 2022 (14) | Non-acute wards and units of 26 public hospitals | One to two visits per patient per week, one hour duration per visit | Completion of two doses (at least 14 days prior) | Negative nucleic acid test (within 48 hours) OR negative rapid antigen test (within 24 hours) | No changing of visitors within a visit. Exemption for recovered within 3 months. |
| 2 | May 31st, 2022 (15) | Acute and specialist hospitals with inpatient services, child and adolescent psychiatric wards/units | One to two visits per patient per week, one hour duration per visit | Completion of required dosage (Vaccine Pass) | Negative nucleic acid test (within 48 hours) OR negative rapid antigen test (within 24 hours) | Excludes ICU, isolation wards, COVID-19 admitting wards. Exemption for recovered within 90 days. |
| Subsequent | March 1st, 2023 (16) | All public hospitals | Subject to ward operations, potential for extended hours | Not specified in detail | Negative rapid antigen test (within 24 hours) | No prior appointment required for most wards. Limit of two visitors at the same time per patient. |

**Table S2.** Details of Hospital SARS-CoV-2 Clusters Sampled for Investigation, including Epidemiological Data and Classification Rationale (May 28 - August 18, 2022).

| **First collection date** | **Last collection date** | **Hospital** | **Ward ID** | **Total cases** | **Staff** | **In-patient** | **Out-patient** | **Lineages** | | **Confirmed nosocomial infection cluster** | **Reasons for exclusion from nosocomial infection /**  **Branch support for confirmed clusters** |
| --- | --- | --- | --- | --- | --- | --- | --- | --- | --- | --- | --- |
| 28-May-2022 | 29-May-2022 | A | R11B* | 2 | 2 | 0 | 0 | BA.2.2 | FALSE | | Inpatients were not involved in transmission |
| 17-Jun-2022 | 26-Jun-2022 | A | AE* | 7 | 6 | 1 | 0 | BA.2.2 | FALSE | | Inpatients were phylogenetically unrelated to other cases |
| 18-Jun-2022 | 22-Jun-2022 | A | E1* | 3 | 0 | 3 | 0 | BA.2.2 (1 case); BA.2.12.1 (2 cases) | TRUE | | 92.4/64 (all 2 tips for BA.2.12.1) |
| 21-Jun-2022 | 23-Jun-2022 | B | B101* | 3 | 0 | 3 | 0 | BA.2.2 | FALSE | | Inpatients were phylogenetically unrelated to other cases |
| 22-Jun-2022 | 25-Jun-2022 | C | 3F | 7 | 2 | 5 | 0 | BA.2.2 | TRUE | | 92.4/64 (all 7 tips) |
| 22-Jun-2022 | 24-Jun-2022 | D | J6* | 2 | 0 | 2 | 0 | BA.2.2 | TRUE | | 77.3/66 (all 2 tips) |
| 27-Jun-2022 | 6-Jul-2022 | E | 11A | 6 | 3 | 3 | 0 | BA.2.2 | TRUE | | 91.1/89 (4 of 6 tips) |
| 28-Jun-2022 | 5-Jul-2022 | F | 3B | 5 | 0 | 5 | 0 | BA.2.2 | TRUE | | 91.1/89 (all 5 tips) |
| 28-Jun-2022 | 4-Jul-2022 | G | CATERING* | 4 | 4 | 0 | 0 | BA.2.2 | FALSE | | Inpatients were not involved in transmission |
| 4-Jul-2022 | 10-Jul-2022 | H | E1&E2&E3 | 9 | 7 | 2 | 0 | BA.2.2 (8 cases); BA.2.12.1 (1 case) | TRUE | | 91.1/89 (all 8 tips for BA.2.2) |
| 4-Jul-2022 | 10-Jul-2022 | G | E5 | 10 | 2 | 7 | 1 | BA.2.2 | TRUE | | 85.7/63 (all 10 tips) |
| Around 5-Jul-2022 | Around 5-Jul-2022 | I | E002 | 7 | 3 | 4 | 0 | BA.2.2 | TRUE | | 85.2/78 (all 7 tips) |
| 7-Jul-2022 | Around 7-Jul-2022 | G | E1 | 2 | 0 | 2 | 0 | BA.2.2 | TRUE | | 78.1/85 (all 2 tips) |
| Around 8-Jul-2022 | Around 8-Jul-2022 | C | 12C* | 3 | 3 | 0 | 0 | BA.2.2 | FALSE | | Inpatients were not involved in transmission |
| 9-Jul-2022 | 14-Jul-2022 | J | STAFF* | 4 | 4 | 0 | 0 | BA.2.2 | FALSE | | Inpatients were not involved in transmission |
| 11-Jul-2022 | 11-Jul-2022 | K | A2R | 2 | 0 | 2 | 0 | BA.2.2 | TRUE | | 92/87 (all 2 tips) |
| 11-Jul-2022 | 19-Jul-2022 | L | 2A* | 2 | 1 | 1 | 0 | BA.2.2 | FALSE | | Inpatients were phylogenetically unrelated to other cases |
| 13-Jul-2022 | 15-Jul-2022 | M | 2DL | 3 | 0 | 2 | 1 | BA.2.2 | TRUE | | 92.9/96 (all 3 tips) |
| 16-Jul-2022 | Around 16-Jul-2022 | J | 3H | 8 | 3 | 5 | 0 | BA.2.2 | TRUE | | 94.6/97 (all 8 tips) |
| 17-Jul-2022 | 18-Jul-2022 | J | AE01* | 2 | 2 | 0 | 0 | BA.2.2 | FALSE | | Inpatients were not involved in transmission |
| Around 22-Jul-2022 | Around 22-Jul-2022 | A | D4 | 9 | 5 | 4 | 0 | BA.2.2 (8 cases); BA.2.12.1 (1 case) | TRUE | | 79.5/75 (all 8 tips for BA.2.2) |
| 22-Jul-2022 | 25-Jul-2022 | N | 9D | 9 | 6 | 3 | 0 | BA.2.2 | TRUE | | 92.4/62 (all 9 tips) |
| 23-Jul-2022 | 10-Aug-2022 | O | L2 | 3 | 0 | 1 | 2 | BA.2.2 | FALSE | | Inpatients were phylogenetically unrelated to other cases |
| 24-Jul-2022 | 27-Jul-2022 | P | C7* | 2 | 0 | 2 | 0 | BA.2.2 | FALSE | | Inpatients were phylogenetically unrelated to other cases |
| 26-Jul-2022 | 28-Jul-2022 | D | E8* | 2 | 0 | 2 | 0 | BA.2.2 | TRUE | | 91.1/89 (all 2 tips) |
| 26-Jul-2022 | 30-Jul-2022 | Q | PMH | 6 | 1 | 5 | 0 | BA.2.2 | TRUE | | 95.3/94 (all 6 tips) |
| 26-Jul-2022 | 2-Aug-2022 | N | NEATS* | 4 | 1 | 3 | 0 | BA.2.2 (3 cases); BA.2.12.1 (1 case) | FALSE | | Inpatients were phylogenetically unrelated to other cases |
| 7-Aug-2022 | 18-Aug-2022 | I | A102 | 22 | 1 | 21 | 0 | BA.5.6 | TRUE | | 82.7/64 (all 22 tips) |
| 12-Aug-2022 | 16-Aug-2022 | A | H1 | 14 | 4 | 10 | 0 | BA.2.2 | TRUE | | 91.1/89 (all 14 tips) |

**Table S3.** Summary of Nosocomial SARS-CoV-2 Infections Officially Reported by the Hong Kong Hospital Authority (May 28 - August 18, 2022). (No mapping is provided between named HA press items and the anonymized A–Q codes.)

| **Earliest reported date** | **Hospital/clinic** | **Sampled in our study** | **Nosocomial infections-patient** | **Nosocomial infections-staff** |
| --- | --- | --- | --- | --- |
| 2022-06-04 | Queen Mary Hospital |  | 1 | 1 |
| 2022-06-22 | Prince of Wales Hospital | Yes | 5 | 2 |
| 2022-06-27 | Tseung Kwan O Hospital | Yes | 3 | - |
| 2022-07-03 | Caritas Medical Centre | Yes | 3 | - |
| 2022-07-04 | Alice Ho Miu Ling Nethersole Hospital | Yes | 3 |  |
| 2022-07-04 | Alice Ho Miu Ling Nethersole Hospital | Yes | 9 | 1 |
| 2022-07-04 | Castle Peak Hospital | Yes | 3 | - |
| 2022-07-05 | Castle Peak Hospital |  | 1 | - |
| 2022-07-05 | Hong Kong Buddhist Hospital |  | 16 | 4 |
| 2022-07-07 | Pok Oi Hospital | Yes | 2 | 4 |
| 2022-07-08 | Kowloon Hospital |  | 1 | - |
| 2022-07-08 | Tung Wah Eastern Hospital | Yes | 2 | 1 |
| 2022-07-10 | Queen Elizabeth Hospital |  | 2 | 2 |
| 2022-07-10 | Kwai Chung Hospital |  | 1 | - |
| 2022-07-11 | Alice Ho Miu Ling Nethersole Hospital |  | - | 1 |
| 2022-07-11 | Castle Peak Hospital |  | - | 1 |
| 2022-07-13 | Hong Kong Buddhist Hospital |  | 13 | 5 |
| 2022-07-13 | Tai Po Hospital | Yes | 3 | 2 |
| 2022-07-14 | Queen Elizabeth Hospital |  | 6 | 3 |
| 2022-07-15 | North District Hospital | Yes | 4 | 6 |
| 2022-07-17 | Kowloon Hospital |  | 8 | 1 |
| 2022-07-18 | Tuen Mun Hospital | Yes | 4 | 4 |
| 2022-07-18 | Kowloon Hospital |  | 3 | 1 |
| 2022-07-19 | North District Hospital |  | 1 | - |
| 2022-07-20 | North District Hospital |  | 1 | - |
| 2022-07-20 | Kwai Chung Hospital |  | 4 | 1 |
| 2022-07-23 | Shatin Hospital | Yes | 4 | 4 |
| 2022-07-24 | Kowloon Hospital |  | - | 1 |
| 2022-07-24 | Kwai Chung Hospital |  | 1 | - |
| 2022-07-24 | Queen Mary Hospital |  | 3 | 4 |
| 2022-07-25 | Kowloon Hospital |  | 1 | - |
| 2022-07-25 | Tuen Mun Hospital |  | 1 | - |
| 2022-07-26 | Kowloon Hospital |  | 1 | - |
| 2022-07-26 | Shatin Hospital |  | - | 1 |
| 2022-07-26 | Princess Margaret Hospital | Yes | 5 | 1 |
| 2022-07-27 | Shatin Hospital |  | - | 1 |
| 2022-07-29 | Kwai Chung Hospital |  | 1 | - |
| 2022-08-03 | TWGHs Wong Tai Sin Hospital |  | 4 | - |
| 2022-08-04 | Castle Peak Hospital | Yes | 15 | 2 |
| 2022-08-05 | TWGHs Wong Tai Sin Hospital |  | - | 1 |
| 2022-08-08 | Kwai Chung Hospital |  | 1 | - |
| 2022-08-10 | Castle Peak Hospital |  | 1 | - |
| 2022-08-11 | Castle Peak Hospital |  | 2 | - |
| 2022-08-11 | Tuen Mun Hospital | Yes | 9 | 5 |
| 2022-08-13 | Pamela Youde Nethersole Eastern Hospital |  | 6 | 1 |
| 2022-08-13 | Kwai Chung Hospital |  | 2 | - |
| 2022-08-13 | Castle Peak Hospital |  | 2 | - |
| 2022-08-14 | Castle Peak Hospital |  | 3 | - |
| 2022-08-16 | Castle Peak Hospital |  | 1 | - |
| 2022-08-17 | Pamela Youde Nethersole Eastern Hospital |  | 2 | - |
| 2022-08-18 | Pamela Youde Nethersole Eastern Hospital |  | 1 | 1 |

**Table S4.** Generalized Additive Models (GAM) model results summary.

| **Predictor** | **Estimate (log)** | **SE** | **Z value** | **P value** | **IRR** | **IRR CI_lower** | **IRR CI_upper** |
| --- | --- | --- | --- | --- | --- | --- | --- |
| data_local_transport_total | 0.001 | 0.002 | 0.345 | 0.73 | 1.001 | 0.997 | 1.004 |
| cross_border_Airport_Arrival | 0 | 0 | 0.06 | 0.952 | 1 | 0.999 | 1.001 |
| cross_border_Airport_Departure | 0 | 0 | -0.264 | 0.792 | 1 | 0.999 | 1 |
| cross_border_Mainland_border_Arrival | 0 | 0.001 | -0.316 | 0.752 | 1 | 0.999 | 1.001 |
| cross_border_Mainland_border_Departure | -0.001 | 0.001 | -0.646 | 0.518 | 0.999 | 0.997 | 1.001 |
| google_index_Public_Parks | -0.019 | 0.031 | -0.611 | 0.541 | 0.981 | 0.923 | 1.043 |
| google_index_Public_Grocery_and_pharmacy | 0.005 | 0.027 | 0.181 | 0.857 | 1.005 | 0.953 | 1.06 |
| google_index_Private_Workplaces | 0.002 | 0.014 | 0.18 | 0.857 | 1.002 | 0.976 | 1.03 |

**Figure S1. The distribution of admission-to-positive interval (API) for inpatient cases in this study.** The inner plot is a zoom view of the case distribution between API of 0 to 80.


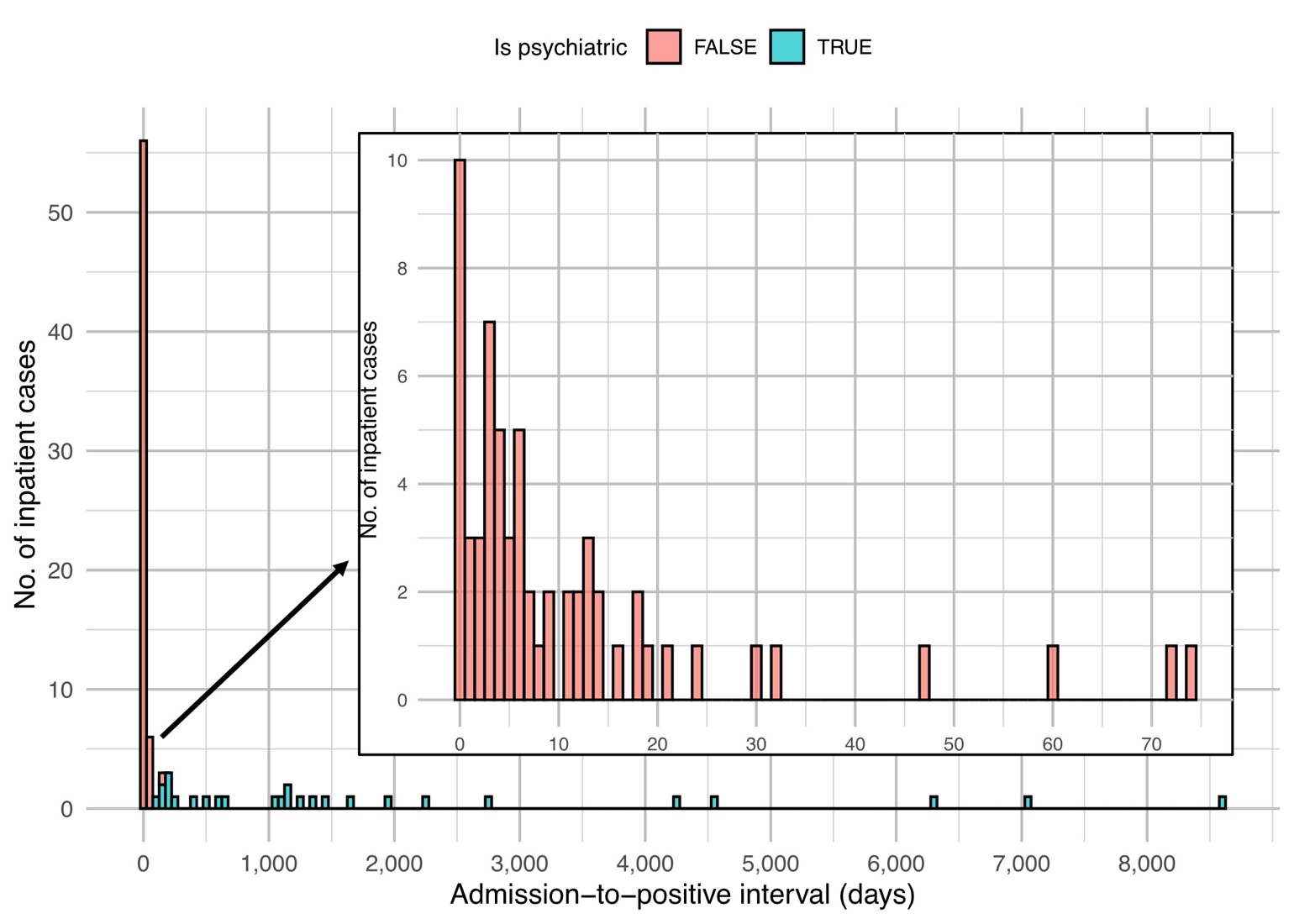


**Figure S2.** Extracted clade from the full reconstructed BA.2.2 and BA.2.12.1 maximum likelihood phylogenetic trees, focusing on sequences from the Hospital A. Focused tips are labelled; Branch supports for ancestral nodes of focused sequences are labeled in blue.

**Figure S3.** Extracted clade from the full reconstructed BA.2.2 maximum likelihood phylogenetic tree, focusing on sequences from the Hospital B. Focused tips are labelled; Branch supports for ancestral nodes of focused sequences are labeled in blue.

**Figure S4.** Extracted clade from the full reconstructed BA.2.2 maximum likelihood phylogenetic tree, focusing on sequences from the Hospital C. Focused tips are labelled; Branch supports for ancestral nodes of focused sequences are labeled in blue.

**Figure S5.** Extracted clade from the full reconstructed BA.2.2 maximum likelihood phylogenetic tree, focusing on sequences from the Hospital D. Focused tips are labelled; Branch supports for ancestral nodes of focused sequences are labeled in blue.

**Figure S6.** Extracted clade from the full reconstructed BA.2.2 maximum likelihood phylogenetic tree, focusing on sequences from the Hospital E. Focused tips are labelled; Branch supports for ancestral nodes of focused sequences are labeled in blue.

**Figure S7.** Extracted clade from the full reconstructed BA.2.2 maximum likelihood phylogenetic tree, focusing on sequences from the Hospital F. Focused tips are labelled; Branch supports for ancestral nodes of focused sequences are labeled in blue.

**Figure S8.** Extracted clade from the full reconstructed BA.2.2 maximum likelihood phylogenetic tree, focusing on sequences from the Hospital G. Focused tips are labelled; Branch supports for ancestral nodes of focused sequences are labeled in blue.

**Figure S9.** Extracted clade from the full reconstructed BA.2.2 and BA.2.12.1 maximum likelihood phylogenetic trees, focusing on sequences from the Hospital H. Focused tips are labelled; Branch supports for ancestral nodes of focused sequences are labeled in blue.

**Figure S10.** Extracted clade from the full reconstructed BA.2.2 and BA.5.6 maximum likelihood phylogenetic trees, focusing on sequences from the Hospital I. Focused tips are labelled; Branch supports for ancestral nodes of focused sequences are labeled in blue.

**Figure S11.** Extracted clade from the full reconstructed BA.2.2 maximum likelihood phylogenetic tree, focusing on sequences from the Hospital J. Focused tips are labelled; Branch supports for ancestral nodes of focused sequences are labeled in blue.

**Figure S12.** Extracted clade from the full reconstructed BA.2.2 maximum likelihood phylogenetic tree, focusing on sequences from the Hospital K. Focused tips are labelled; Branch supports for ancestral nodes of focused sequences are labeled in blue.

**Figure S13.** Extracted clade from the full reconstructed BA.2.2 maximum likelihood phylogenetic tree, focusing on sequences from the Hospital L. Focused tips are labelled; Branch supports for ancestral nodes of focused sequences are labeled in blue.

**Figure S14.** Extracted clade from the full reconstructed BA.2.2 maximum likelihood phylogenetic tree, focusing on sequences from the Hospital M. Focused tips are labelled; Branch supports for ancestral nodes of focused sequences are labeled in blue.

**Figure S15.** Extracted clade from the full reconstructed BA.2.2 and BA.2.12.1 maximum likelihood phylogenetic trees, focusing on sequences from the Hospital N. Focused tips are labelled; Branch supports for ancestral nodes of focused sequences are labeled in blue.

**Figure S16.** Extracted clade from the full reconstructed BA.2.2 maximum likelihood phylogenetic tree, focusing on sequences from the Hospital O. Focused tips are labelled; Branch supports for ancestral nodes of focused sequences are labeled in blue.

**Figure S17.** Extracted clade from the full reconstructed BA.2.2 maximum likelihood phylogenetic tree, focusing on sequences from the Hospital P. Focused tips are labelled; Branch supports for ancestral nodes of focused sequences are labeled in blue.

**Figure S18.** Extracted clade from the full reconstructed BA.2.2 maximum likelihood phylogenetic tree, focusing on sequences from the Hospital Q. Focused tips are labelled; Branch supports for ancestral nodes of focused sequences are labeled in blue.

**Figure S19.** Estimated smooth function of the GAM model with the fitted data.

**References**

1. Wong RC, Wong AH, Ho YI, Leung EC, Lai RW. Evaluation on testing of deep throat saliva and lower respiratory tract specimens with Xpert Xpress SARS-CoV-2 assay. J Clin Virol. 2020;131:104593. Epub 20200816. doi: 10.1016/j.jcv.2020.104593. PubMed PMID: 32823131; PubMed Central PMCID: PMCPMC7429071.

2. Shu Y, McCauley J. GISAID: Global initiative on sharing all influenza data–from vision to reality. Eurosurveillance. 2017;22(13):30494.

3. Katoh K, Standley DM. MAFFT multiple sequence alignment software version 7: improvements in performance and usability. Mol Biol Evol. 2013;30(4):772-80. Epub 20130116. doi: 10.1093/molbev/mst010. PubMed PMID: 23329690; PubMed Central PMCID: PMCPMC3603318.

4. Minh BQ, Schmidt HA, Chernomor O, Schrempf D, Woodhams MD, von Haeseler A, et al. IQ-TREE 2: New Models and Efficient Methods for Phylogenetic Inference in the Genomic Era. Mol Biol Evol. 2020;37(5):1530-4. doi: 10.1093/molbev/msaa015. PubMed PMID: 32011700; PubMed Central PMCID: PMCPMC7182206.

5. Hoang DT, Chernomor O, von Haeseler A, Minh BQ, Vinh LS. UFBoot2: Improving the Ultrafast Bootstrap Approximation. Molecular Biology and Evolution. 2018;35(2):518-22. doi: 10.1093/molbev/msx281.

6. Guindon S, Dufayard J-F, Lefort V, Anisimova M, Hordijk W, Gascuel O. New Algorithms and Methods to Estimate Maximum-Likelihood Phylogenies: Assessing the Performance of PhyML 3.0. Systematic Biology. 2010;59(3):307-21. doi: 10.1093/sysbio/syq010.

7. Sagulenko P, Puller V, Neher RA. TreeTime: Maximum-likelihood phylodynamic analysis. Virus Evol. 2018;4(1):vex042. Epub 20180108. doi: 10.1093/ve/vex042. PubMed PMID: 29340210; PubMed Central PMCID: PMCPMC5758920.

8. Rambaut A, Lam TT, Max Carvalho L, Pybus OG. Exploring the temporal structure of heterochronous sequences using TempEst (formerly Path-O-Gen). Virus Evol. 2016;2(1):vew007. Epub 20160409. doi: 10.1093/ve/vew007. PubMed PMID: 27774300; PubMed Central PMCID: PMCPMC4989882.

9. Suchard MA, Lemey P, Baele G, Ayres DL, Drummond AJ, Rambaut A. Bayesian phylogenetic and phylodynamic data integration using BEAST 1.10. Virus Evol. 2018;4(1):vey016. Epub 20180608. doi: 10.1093/ve/vey016. PubMed PMID: 29942656; PubMed Central PMCID: PMCPMC6007674.

10. Fonseca PLC, Moreira FRR, de Souza RM, Guimaraes NR, Carvalho NO, Adelino TER, et al. Tracking the turnover of SARS-CoV-2 VOCs Gamma to Delta in a Brazilian state (Minas Gerais) with a high-vaccination status. Virus Evol. 2022;8(2):veac064. Epub 20220727. doi: 10.1093/ve/veac064. PubMed PMID: 35996592; PubMed Central PMCID: PMCPMC9384558.

11. Didelot X, Siveroni I, Volz EM. Additive Uncorrelated Relaxed Clock Models for the Dating of Genomic Epidemiology Phylogenies. Mol Biol Evol. 2021;38(1):307-17. doi: 10.1093/molbev/msaa193. PubMed PMID: 32722797; PubMed Central PMCID: PMCPMC8480190.

12. Gill MS, Lemey P, Faria NR, Rambaut A, Shapiro B, Suchard MA. Improving Bayesian population dynamics inference: a coalescent-based model for multiple loci. Mol Biol Evol. 2013;30(3):713-24. Epub 20121122. doi: 10.1093/molbev/mss265. PubMed PMID: 23180580; PubMed Central PMCID: PMCPMC3563973.

13. Lemey P, Ruktanonchai N, Hong SL, Colizza V, Poletto C, Van den Broeck F, et al. Untangling introductions and persistence in COVID-19 resurgence in Europe. Nature. 2021;595(7869):713-7.

14. Government Information Services HKSAR. Government adjusts visiting arrangements for public hospitals: [Press release]; [updated 2022/05/03]. Available from: <https://www.info.gov.hk/gia/general/202205/03/P2022050300529.htm>.

15. Government Information Services HKSAR. Government further adjusts visiting arrangements for public hospitals and residential care homes for the elderly and persons with disabilities: [Press release]; [updated 2022/05/26]. Available from: <https://www.info.gov.hk/gia/general/202205/26/P2022052600625.htm>.

16. news.gov.hk. HA & residential care homes visiting curbs to be lifted: [News Article]; [updated 2023/02/27]. Available from: <https://www.news.gov.hk/eng/2023/02/20230227/20230227_155329_832.html>.
